# Isolation of *F. novicida*-Containing Phagosome from Infected Human Monocyte Derived Macrophages

**DOI:** 10.3389/fcimb.2017.00303

**Published:** 2017-07-05

**Authors:** Valentina Marecic, Olga Shevchuk, Mateja Ozanic, Mirna Mihelcic, Michael Steinert, Antonija Jurak Begonja, Yousef Abu Kwaik, Marina Santic

**Affiliations:** ^1^Department of Microbiology and Parasitology, Faculty of Medicine, University of RijekaRijeka, Croatia; ^2^Department of Microbiology, Institut für Mikrobiologie, Technische Universität Braunschweig and Helmholtz Center for Infection ResearchBraunschweig, Germany; ^3^Department of Biotechnology, University of RijekaRijeka, Croatia; ^4^Department of Microbiology and Immunology and Center for Predictive MedicineLouisville, KY, United States

**Keywords:** phagocytosis, organelle purification, pathogen-containing phagosomes, *Francisella*, human macrophages

## Abstract

*Francisella* is a gram-negative bacterial pathogen, which causes tularemia in humans and animals. A crucial step of *Francisella* infection is its invasion of macrophage cells. Biogenesis of the *Francisella*-containing phagosome (FCP) is arrested for ~15 min at the endosomal stage, followed by gradual bacterial escape into the cytosol, where the microbe proliferates. The crucial step in pathogenesis of tularemia is short and transient presence of the bacterium within phagosome. Isolation of FCPs for further studies has been challenging due to the short period of time of bacterial residence in it and the characteristics of the FCP. Here, we will for the first time present the method for isolation of the FCPs from infected human monocytes-derived macrophages (hMDMs). For elimination of lysosomal compartment these organelles were pre-loaded with dextran coated colloidal iron particles prior infection and eliminated by magnetic separation of the post-nuclear supernatant (PNS). We encountered the challenge that mitochondria has similar density to the FCP. To separate the FCP in the PNS from mitochondria, we utilized iodophenylnitrophenyltetrazolium, which is converted by the mitochondrial succinate dehydrogenase into formazan, leading to increased density of the mitochondria and allowing separation by the discontinuous sucrose density gradient ultracentrifugation. The purity of the FCP preparation and its acquisition of early endosomal markers was confirmed by Western blots, confocal and transmission electron microscopy. Our strategy to isolate highly pure FCPs from macrophages should facilitate studies on the FCP and its biogenesis.

## Introduction

Intracellular bacteria invade eukaryotic cells, followed by subversion of endocytic pathway, which results in formation of membrane-bound phagosomes. They are cable to modulate the membrane protein and lipid composition of phagosomes. This modulation is crucial for bacterial survival within the host cell because it either promotes the establishment of an intact replicative niche or allow the pathogen to escape to replication-permissive cytosol. Many intracellular bacterial pathogens have unique life cycles. Cytosolic bacteria, like *Shigella* (Ray et al., 2010) and *Listeria* (Camejo et al., 2011) modulate the endosomal-lysosomal membrane-bound compartments and escape into the cytosol, which provides environment rich in nutrients. Intracellular bacteria, *Salmonella* (Steele-Mortimer, 2008; Malik-Kale et al., 2011), *Legionella* (Kagan and Roy, 2002; Shin and Roy, 2008; Isberg et al., 2009), and *Mycobacterium* spp. (Vergne et al., 2004) are intracellular bacterial pathogens that reside and replicate within host endomembrane system. These bacteria overcome the stressful conditions in membrane-bound vacuoles.

*Francisella tularensis* is a gram-negative, highly infectious bacterium. The bacterium causes the zoonotic disease tularemia. *F. tularensis* type A is a dangerous pathogen that constantly raises attention due to potential use as biological weapon. Interestingly, *Francisella novicida* shares many similarities to type A strain due to genome sequence, intracellular life cycle and infectivity.

*F. tularensis* can invade and multiply within a range of cell types (Buddingh and Womack, 1941; Shepard, 1959; Anthony L. S. et al., 1991; Ben Nasr et al., 2006; Lindemann et al., 2007), but *in vivo* its primary target are macrophages (Fortier et al., 1994). Within mammalian and arthropod-derived cells, *Francisella* resides in acidic vacuole prior to escape to the cytosol, where it replicates (Santic et al., 2009; Llewellyn et al., 2011). In contrast, within amoeba cells bacterium resides and replicates within non-acidified, membrane-bound vacuoles (Lauriano et al., 2004; Santic et al., 2008).

After entry, it is enclosed within a unique compartment. Intracellular proliferation is essential for *Francisella* virulence, and a lot of effort has been made on understanding of specific steps in the intracellular cycle of this bacterium. *Francisella* survival and proliferation strategies rely on entering in the initial phagosome along the endocytic pathway and physical escape to the cell cytosol, making this bacterium a typical cytosol-dwelling pathogen (Celli and Zahrt, 2013). Despite the fact that *Francisella* replicates in the cytosol of infected cells, short presence of the *Francisella* in the phagosome is necessary for productive multiplication.

Macrophage infection by *Francisella* begins with initial bacterial recognition at the cell membrane (Clemens et al., 2005). *Francisella* enters into macrophages by looping phagocytosis through cholesterol-rich membrane domains called “lipid rafts” with caveolin-1 (Clemens et al., 2004; Tamilselvam and Daefler, 2008; Moreau and Mann, 2013). Following uptake, *Francisella* resides within an initial vacuolar compartment, the *Francisella*-containing phagosome (FCP). Lipid raft-associated components are incorporated into the FCP during entry and the initial phase of intracellular infection of host cells (Tamilselvam and Daefler, 2008). Cholesterol, as a key structural and regulatory element for the integrity of lipid rafts, has an important role in *Francisella* internalization into macrophages (Tamilselvam and Daefler, 2008). FCP matures to an early endosome state regulated by Rab5, a protein that is critical for endosome-phagosome tethering and fusion (Alvarez-Dominguez et al., 1996; Jahraus et al., 1998; Duclos et al., 2000). The FCP consequently acquires late endosomal markers including CD63, LAMP-1, LAMP-2, and Rab7 (Clemens et al., 2004, 2009; Santic et al., 2005; Checroun et al., 2006; Chong et al., 2008; Wehrly et al., 2009). Eventually the late endosome becomes acidified upon acquisition of the proton vATPase pump that imports hydrogen protons into the vacuole (Chong et al., 2008; Santic et al., 2008). The FCP does not accumulate any lysosomal markers, such as Cathepsin D, or lysosomal tracers (Anthony L. D. et al., 1991; Clemens et al., 2004; Santic et al., 2005; Bonquist et al., 2008). In order to evade lysosome-mediated killing, *Francisella* escapes from the FCP to the cytosol. Vacuolar escape by various strains of *F. tularensis* and *F. novicida* in macrophages and other cells types has been described (Golovliov et al., 2003; Clemens et al., 2004; Santic et al., 2005, 2008; Chong et al., 2008). However, the study of *Francisella* vacuolar escape kinetics has brought controversy to the field varying from the 15 min to 8 h post-infection (Golovliov et al., 2003; Clemens et al., 2004; Santic et al., 2005, 2008; Checroun et al., 2006; McCaffrey and Allen, 2006). However, these differences are likely due to variation in the host cells used, the *Francisella* species and the methodological approaches used by various studies (Golovliov et al., 2003; Clemens et al., 2004; Santic et al., 2005, 2008; Checroun et al., 2006; McCaffrey and Allen, 2006).

The survival and replication of *Francisella* in host cells depends on the expression of the *Francisella* pathogenicity island (FPI) proteins. FPI-deficient mutants fail in formation and maturation of FCP (Nano et al., 2004; Telepnev et al., 2005; McCaffrey and Allen, 2006; Qin and Mann, 2006; Chong et al., 2008, 2013; Broms et al., 2010; Napier et al., 2012; Steele et al., 2013). Moreover, inactivated *F. tularensis*, as well as FPI mutants *iglC* and *pdpC* are not capable to avoid the FCP suggesting that vacuolar escape is a *Francisella* mediated process. This was clearly demonstrated when vacuolar escape deficient mutant *pdpC* was paired on magnetic bead with wild type bacteria. Wild type *F. novicida* secreted effector proteins, which allowed both wild type and *pdpC* to escape the phagosome. Studies have shown that pathogen secrete the IglA-J, PdpA, C, E, DotU, and VgrG into the macrophage cytosol during the infection (Hare and Hueffer, 2014). However, another study shown that IglC, IglI and PdpE, but not IglA and IglG are secreted in a T6SS-dependent manner during infection (Broms et al., 2012). Another importance of phagosome formation is shown by the reduced ability of *Francisella* LVS (live vaccine strain) strain to grow in host cell cytosol after microinjection (Meyer et al., 2015). The brief time spent in the phagosome is a dynamic step during which *Francisella* must actively evade host antimicrobial defenses (Jones et al., 2012). *Francisella* phagosomal escape is requisite to intracellular proliferation and its essential in the *Francisella* intracellular life cycle (Lindgren et al., 2004; Santic et al., 2005; Bonquist et al., 2008; Barker et al., 2009; Wehrly et al., 2009; Broms et al., 2012).

Techniques for the isolation and analysis of phagosomes are important experimental tools in endocytosis and apoptosis research. Since 1969, most of the available methods are based on density gradient ultracentrifugation (Wetzel and Korn, 1969). Here we present a method for isolation of FCP from infected human monocyte-derived macrophages (hMDMs). The method is based on infection of human macrophages with *F. novicida*, followed by mechanical lysis and separation of intracellular organelles. Several adaptations of previously described method are included (Shevchuk et al., 2009; Shevchuk and Steinert, 2013). For elimination of lysosomal compartment these organelles were loaded with dextran coated colloidal iron particles prior infection and eliminated by magnetic separation of post nuclear supernatant (PNS). The treatment of PNS with iodophenylnitrophenyltetrazolium (INT) salt was necessary to increase the density of mitochondria and fractionate it from FCP in a discontinuous sucrose density gradient.

## Materials and methods

### Cultivation of *F. novicida*

*F. novicida* (Birdsell et al., 2009) was grown on buffered charcoal-yeast extract (BCYE) agar at 37°C with 5% CO_2_ atmosphere for 24 h.

### Preparation of hMDMs from blood of healthy human donors

Human monocyte derived macrophages were differentiated from peripheral blood monocytes of healthy volunteers with no history of tularemia. Blood was diluted in ratio 1:2 with 0.9% saline, and 15 ml was applied on top of 7.5 ml of Ficoll-Hypaque (Ficoll-Paque; Pharmacia Fine Chemicals, USA). After 25 min of centrifugation at 300 × g, at room temperature (RT) the layer containing the mononuclear cell fraction was aspirated, transferred to a new tubes and centrifuged for additional 10 min at 300 × g at RT. Obtained monocytes were washed twice with 25 ml of 0.9% saline, resuspended in RPMI with glutamine (Bio Whittaker, Lonza, USA) supplemented with 20% FBS (Invitrogen, USA), and distributed in 6-well ultra-low attachment plates (Cornig Life Sciences, USA). Serum starvation was performed to promote monocyte differentiation to macrophages (Santic et al., 2005; Ozanic et al., 2016). After 3 days of incubation at 37°C in 5% CO_2_ the medium was replaced with 10% FBS RPMI, followed by replacement with 5% FBS RPMI (at day 6). At day 7 cells were scraped, collected and resuspended to desired concentration in RPMI with 1% FBS.

### Preparation of colloidal iron particles

Dextran—coated colloidal iron particles were prepared as follows. Equal volumes of 1.2 M FeCl_2_ (10 ml) and 1.8 M FeCl_3_ (10 ml) were mixed and agitated extensively while adding the same volume (10 ml) of 25% NH_3_ dropwise. The suspension was divided in 5 ml aliquots and placed on a magnetic unit (Dynal, Thermo Fisher, USA). Precipitate was than collected on the bottom of the tubes and washed once with 5% NH_3_, twice with _dd_H_2_O and resuspend in 80 ml of 0.3 M HCl. Solution was magnetically stirred for 30 min. Dextran (4 g, 64 to 76 kDa, Sigma-Aldrich, USA) was added and solution was stirred for further 30 min. In study of distribution of colloidal iron particles within endosomal compartments, small aliquot of prepared colloidal iron was incubated with dextran-tetramethylrhodamine (1 mg/ml, 70 kDa, Sigma-Aldrich, USA) and stirred for 30 min. The samples were dialyzed against 5 l of cold water, changing water four times during 2 days. The final suspension was filtered through filter paper and was used immediately or stored at 4°C for maximum 3 months. The concentration of obtained iron solution was ~10 mg/ml (Rodriguez-Paris et al., 1993).

### Preparation of OptiPrep™ density gradients

OptiPrep gradients were prepared by mixing of two working solutions, 10 and 45% of OptiPrep (Sigma-Aldrich, USA) in HB buffer (0.5 mM Na_2_EGTA, 20 mM HEPES, 250 mM Sucrose) in gradient mixer (Model #GM-40; C.B.S. Scientific Co, USA). Gradients were poured into polyallomer centrifuge tubes (9/16 × 3-3/4″; 14 × 95 mm; Beckman Coulter, USA) and used immediately or kept at 4°C overnight.

### Infection of hMDMs with *F. novicida*

A total of 5·10^7^ hMDMs were seeded in 30 ml of RPMI supplemented with 1% FBS in 75 cm^2^ cell culture flasks (TPP, Switzerland). Colloidal iron particles were added to a final concentration of 1 mg/ml, gently distributed 15 min before infection and left in the medium. The cells were infected with *F. novicida* at a multiplicity of infection (MOI) 10. In order to achieve synchronized infection, the cells were centrifuged immediately after infection at 100 × g for 3 min at RT. After 15 min of incubation at 37°C the cells were scraped, transferred to a 50 ml tube and centrifuged at 230 × g for 7 min at 4°C. Cells were washed twice in 30 ml of ice cold PBS and once in 10 ml of ice cold HB buffer.

### Isolation of *F. novicida*-containing phagosome

For the isolation of FCP pellet of infected hMDMs was resuspended in 2 ml of cold HB buffer supplemented with EDTA-free protease inhibitor cocktail (Roche Diagnostic, Penzberg, Germany) according to manufacture protocol and with 5 mg/ml INT (Sigma-Aldrich, USA). The cells were mechanically disrupted in a Dura Grind stainless-steel homogenizer (Dounce Dura-Grind® Tissue Grinder; Braintree, Scientific, Inc.), transferred to a new tube and incubated with Benzonase (50 units/ml, Sigma-Aldrich, USA) for 7 min at 37°C. The nuclear and cell debris were removed by centrifugation at 110 × g for 5 min at 4°C. Obtained PNS was transferred to a new tubes and additional 2 ml of HB buffer with protease inhibitor cocktail was added to remaining pellet, carefully mixed and centrifuged at 100 × g for 3 min at 4°C. PNS was run through the MiniMACS column (OctoMACS™ Separation Unit; Miltenyi Biotec, Germany) to eliminate the lysosomal compartments loaded with colloidal iron. The flow through fraction was carefully applied on top of 8 ml of 10 to 45% OptiPrep gradient and centrifuged for 2 h in SW40 swing Rotor (Beckman Coulter, USA) at 100,000 × g at 4°C. After centrifugation, about 800 μl fractions were carefully collected from the top of gradient with cutted 1 ml tip. To analyze distribution of bacteria in gradient fractions, 10 μl of each fraction was diluted in 190 μl of _dd_H_2_O and plated on BCYE square plates 120 × 120 mm (Greiner, Sigma-Aldrich, USA). After 2 days of incubation at 37°C the CFU of *F. novicida* were calculated.

### Confocal laser scanning microscopy

The hMDMs were infected with *F. novicida* at MOI 10. At 15 min after infection the cells were washed with PBS, fixed using 4% paraformaldehyde (PFA, Sigma-Aldrich, USA) for 30 min at 4°C and permeabilized with 0.5% Triton X-100 (Sigma-Aldrich, USA). The coverslips were incubated with mouse monoclonal anti *Francisella* antibodies (1:5,000), washed with PBS and incubated with Alexa Fluor 555 (1:4,000, Molecular probes, USA) secondary antibodies for 1 h at RT.

To study the integrity of isolated FCP, equal fractions of phagosomes were seeded onto 24-well coverslips and centrifuged at 200 × g for 10 min at 4°C, followed by fixation with 4% PFA for 15 min at RT. Prepared samples were stained with 1 μl/ml of propidium iodide (PI) (Serva, Germany) for 25 min in the dark. Control samples were permeabilized with methanol at −20°C for 5 min.

To study the labeling of endosomal compartments with dextran-tetramethylrhodamine coated colloidal iron, the hMDMs were seeded on coverslips. The cells were loaded with dextran-tetramethylrhodamine colloidal iron for 15 min. After 15 min the colloidal iron particles were left in the medium and the cells were additionally infected with *F. novicida* for 15 min at MOI 10 followed by centrifugation at 100 × g for 3 min at RT. At 15 min after infection, and 30 min of colloidal iron particles load. the cells were washed, fixed and permeabilized as described above. The coverslips were incubated with mouse monoclonal anti *Francisella* antibodies (1:5,000), mouse monoclonal early endosome antigen (EEA1,1:1,000, Bio Rad, USA), mouse monoclonal lysosome associated membrane protein 1 (Lamp-1, 1:1,000, Bio Rad, USA) and mouse monoclonal anti Cathepsin-D (1:1,000, BD Biosciences, USA). The coverslips were washed with PBS and incubated with donkey anti-goat Alexa Fluor 488 and goat anti-mouse Alexa Fluor 647 (1:4,000, Molecular probes, USA) secondary antibodies for 1 h at RT. All samples were mounted in Mowiol 4-88 (Sigma-Aldrich, USA) and analyses were performed on FV 1000 Olympus confocal microscope.

### SDS-PAGE and western blot

For Western blot analysis, equal amount of fraction proteins was applied onto 10% SDS-PAGE. After separation, proteins were transferred to nitrocellulose membrane in Transfer Buffer (Tris Base, Glycine, Methanol, _dd_H_2_O) and blocked for 1 h at room temperature in 1x Tris Buffered Saline (TBS, Sigma-Aldrich, USA) with 0.1% (w/v) Tween-20 (TBST, Sigma-Aldrich, USA) and 3% (m/v) Bovine Serum Albumine (BSA, Sigma-Aldrich, USA). Monoclonal rabbit antibody against human Rab5 (1:1,000, Cell Signaling Technology, USA), rabbit antibody against mitochondrial apoptosis-inducing factor (AIF, 1:1,000, Cell Signaling Technology, USA), mouse monoclonal KDEL antibody (1:100, Santa Cruz Biotechnology, USA), antibody against Golgi matrix protein of 130 kDa (gm130, BD Biosciences, USA), mouse monoclonal EEA1 and Lamp-1 were used for overnight incubation in staining buffer (3% BSA in TBST). After washing three times for 10 min in TBST, secondary anti-rabbit IgG and anti-mouse IgG conjugated horseradish peroxidase antibodies (1:1,000, Cell Signaling Technology, USA) were added for 1 h at RT. Membrane was again washed three times for 10 min in TBST. Enhanced chemiluminescence detection reagents Luminal Enhancer Solution (GE Healthcare, UK) and Peroxide Solution (GE Healthcare, UK) were used for visualization of the detected proteins by Bio Rad Chemi Doc XRR+ (Bio Rad Laboratories, USA).

### Transmission electron microscopy

For transmission electron microscopy, the samples were transferred in 12-well cell culture plates (TPP, Switzerland). The samples were washed with 1x Sorensen buffer (TCS Biosciences Ltd., UK) and fixed using 2.5% glutaraldehyde (SPI Supplies, USA) for 45 min at 4°C. The post fixation was performed with 1% OsO_4_ (SPI Supplies, USA) for 45 min at 4°C. The sample was dehydrated by ethanol series with increased concentration, embedded in epoxy resin (SPI Supplies, USA) and polymerized for 24–48 h at 60°C. Ultra-thin sections were cut and examined by Phillips Morgany transmission electron microscope.

### Statistics

Statistical significances were determined using two-tailed Student's *t*-test. Statistical analyses were performed using Statistica (Statsoft) software version 12 or with GraphPad Prizm version 6.0 software. *P* < 0.001 were accepted as significantly different and were denoted by ^*^.

### Ethics statement

This study was carried out in accordance with the recommendations of Health Care Act Republic of Croatia (NN 158/08, 71/10, 139/10, 22/11, 84/11, 12/12, 35/12, 70/12 i 82/13), Act on the Protection of Patient's rights Republic of Croatia (NN 169/04, 37/08), was approved by the Ethical committee of Clinical Hospital Centre Rijeka as well as Ethical committee of Faculty of Medicine, with written informed consent from all subjects. All subjects gave written informed consent in accordance with the Declaration of Helsinki.

## Results

### *F. novicida*-containing phagosome isolated from infected hMDMs

**Figure 1:**
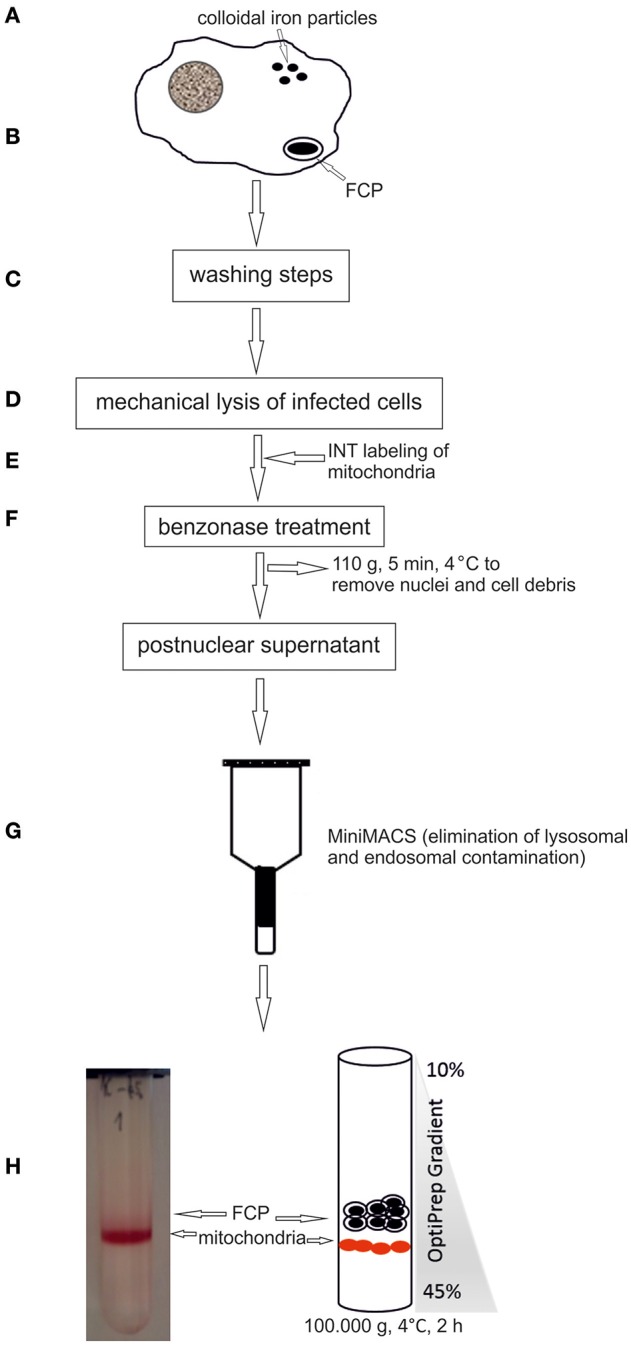
Schematic illustration of the FCPs isolation procedure. **(A)** hMDMs were loaded with colloidal iron particles. **(B)** Cells were infected with *F. novicida*, centrifuged to synchronize the infection, and the infection was allowed to proceed for 15 min. **(C)** Several washing steps were performed. **(D)** The cells were lysed mechanically. **(E)** Mitochondria were labeled with INT. **(F)** For reducing viscosity the suspension was treated with benzonase and centrifuged at low speed to remove cell debris and nuclei. **(G)** Post nuclear supernatant was run through a MiniMACS separation column to eliminate lysosomes. **(H)** The FCP were purified by OptiPrep density gradient ultracentrifugation.

**Figure 2:**
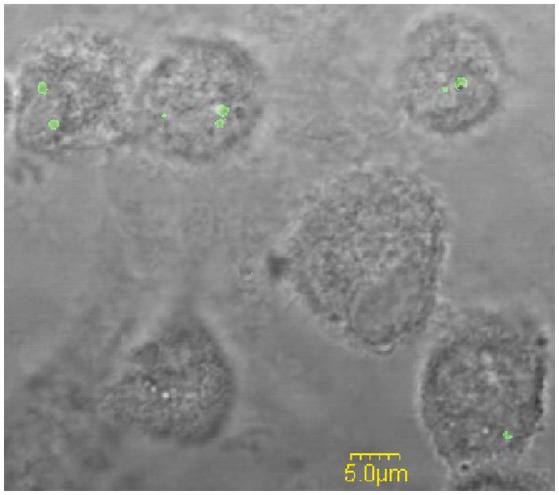
The analysis of the percentage of infected hMDMs. Representative fluorescence microscopy images of hMDMs infected with *F. novicida* at MOI 10 for 15 min. The examination of 100 hMDMs from three different coverslips shows that around 70% of the cells were infected.

**Figure 3:**
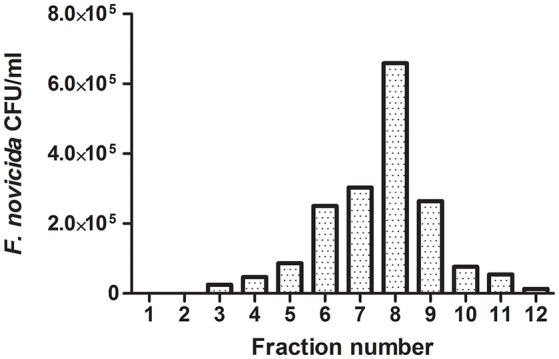
Representative distribution of FCPs in 10–45% OptiPrep gradient fractions. An aliquot of each OptiPrep fraction was plated onto a BCYE-agar plate and CFU/ml of *F. novicida* were counted. The fraction with the highest bacterial number corresponds to FCP fractions.

Following phagocytic uptake, *Francisella* resides within special vacuole-FCP and its formation is absolutely required for intracytoplasmic replication of bacteria (Checroun et al., 2006). Because of apparent importance of this organelle during establishment of infection we optimized the method of FCP isolation (Figure 1). Human macrophages were infected with *F. novicida* at multiplicity of infection 10 resulting in 70% of hMDMs infected with bacteria at 15 min after infection (Figure 2). Macrophages, free of extracellular bacteria were disrupted in a Dura Grind stainless-steel homogenizer by optimized number of strokes. The unbroken cells and nuclei were removed by centrifugation. Obtained PNS was treated with Benzonase, an enzyme mixture for nucleic acid degradation, which allows reduction of sample viscosity and allows the separation of FCP from other organelles in PNS. The distribution of *F. novicida* in the gradient after ultracentrifugation was determined by plating fractions onto BCYE agar plates and counting bacterial CFU/ml (Figure 3). Our results showed that the highest number of *F. novicida* was in fraction 8 and it reached 6.5 × 10^5^ CFU/ml (Figure 3). The fractions with highest number of bacteria were routinely proceeded for further analysis.

### Separation of FCP from subcellular organelles

During isolation of bacterial vacuole, it is crucial to minimalize artifacts caused by other organelles. The successful separation of FCP from subcellular organelles was assessed by Western blot and transmission electron microscopy.

**Figure 4:**
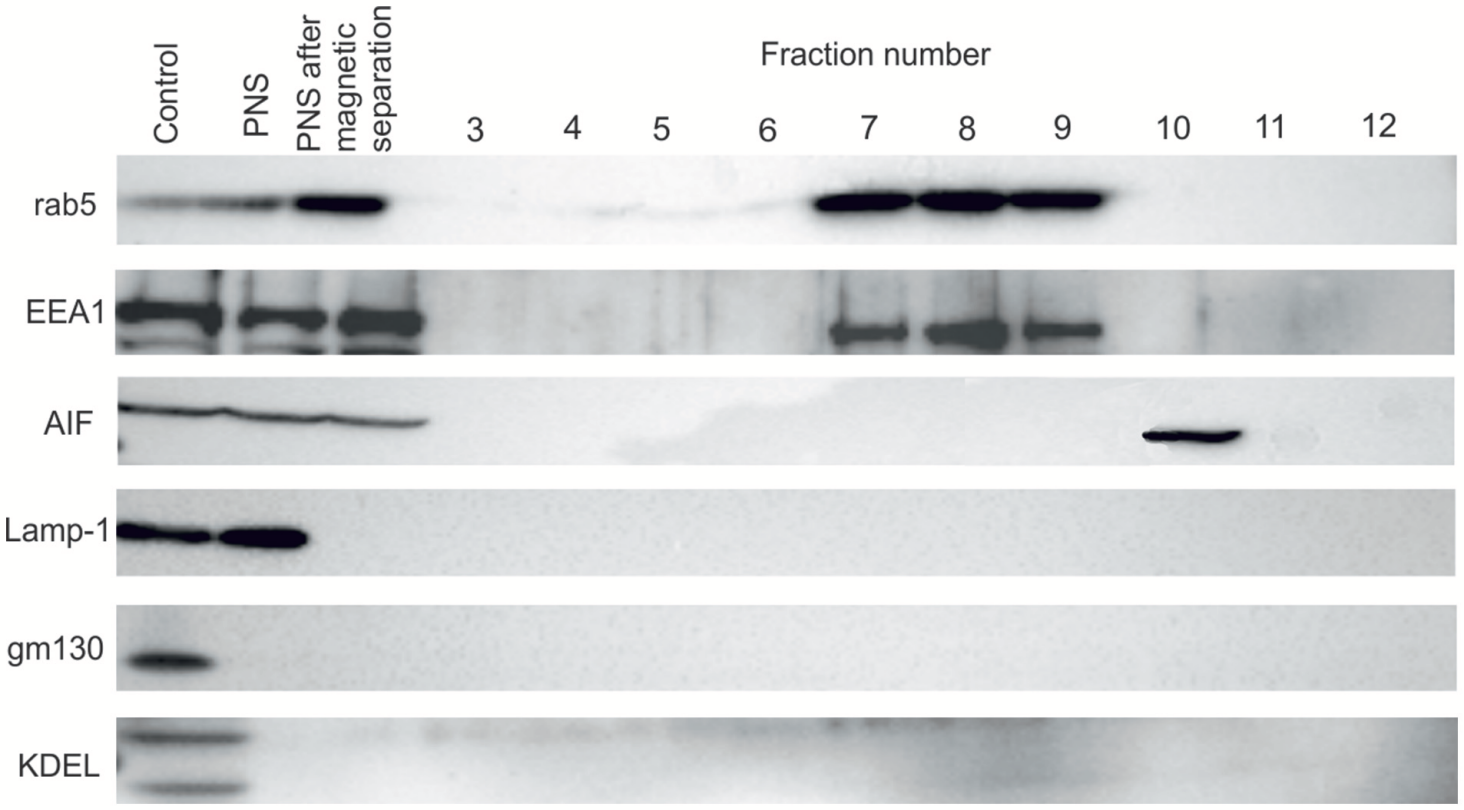
Western blot analysis of OptiPrep fractions. Fractions 3 to 12 as well as PNS before and after magnetic separation were tested using the markers for the following compartments: early endosome (Rab5 and EEA1), lysosomes (Lamp-1), Golgi (gm130), mitochondria (AIF) and ER (KDEL). hMDMs lysate was used as a control.

For separation of FCP from mitochondria we treated the PNS with INT, which results in formation of formazan, a product of activity of mitochondrial succinate dehydrogenase. This step was necessary to increase the density of mitochondria and separate two organelles by ultracentrifugation. The distribution of early endosome markers Rab5 and EEA1, lysosomal marker Lamp-1 as well and mitochondrial marker AIF, Golgi marker gm130 and ER marker KDEL were assessed by WB. Rab5 and EEA1 were enriched in fractions with the highest number of bacteria of the OptiPrep gradient, consistent with the accumulation of FCP in this fraction (Figure 4). AIF was enriched in fraction 10 of the OptiPrep gradient, confirming the presence of mitochondria in these fractions (Figure 4). Obtained results confirmed the separation of FCP from mitochondria by ultracentrifugation. Additionally, in gradient fractions formazan was visible after ultracentrifugation, and could be used as a marker for estimation of localization of bacterial fractions that were above this formazan circle (Figure 1). Further, our results showed that Golgi apparatus and endoplasmic reticulum are eliminated during the purification of the FCP and are not present in gradient fractions (Figure 4). The distribution of Lamp-1 in PNS before and after the magnetic separation, as well as in gradient fractions, was tested by Western blot (Figure 4). The results showed that lysosomes were present in PNS before magnetic separation and eliminated with this procedure.

**Figure 5:**
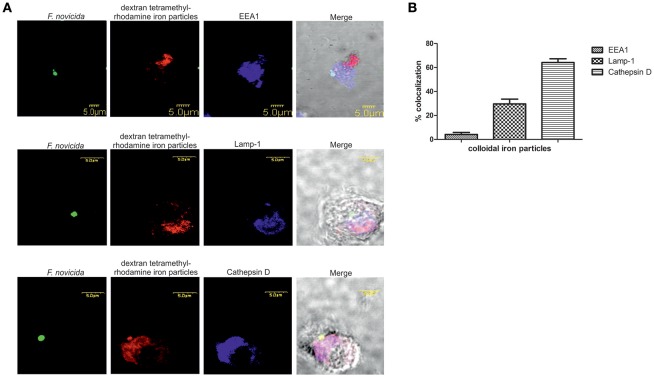
The distribution of dextran-tetramethylrhodamine iron particles within the endocytic pathway in *F. novicida* infection **(A)**. Representative confocal laser scanning microscopy images of colocalization of dextran-tetramethylrhodamine iron particles with Lamp-1, Cathepsin-D, and EEA1 in *F. novicida* infected hMDMs. The images are representatives of 100 infected cells examined from three different cover slips. **(B)** Quantification of colocalization of the colloidal iron particles with EEA1, Lamp-1, and Cathepsin D. The results shown are representative of three independed experiments, and error bars represent standard deviation of triplicate samples.

To investigate the trafficking of the dextran coated colloidal iron particles within the endocytic pathways after infection of hMDMs with *F. novicida* the confocal microscopy was used. Our results showed that around 30% of dextran-tetramethylrhodamine colloidal iron particles colocolized with Lamp-1 and ~65% with Cathepsin-D (Figure 5B). In contrast, *F. novicida* colocalized with EEA1 (Figure 5A), indicating that most of colloidal iron does not interfere with early *F. novicida* phagosome.

**Figure 6:**
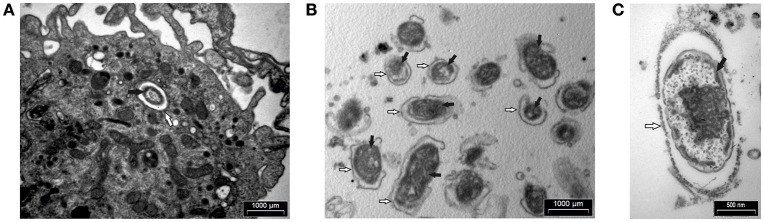
TEM analyses of hMDMs infected with *F. novicida* at MOI 10 at 15 min after infection **(A)** and isolated FCP **(B,C)**. Samples were washed and fixed with glutaraldehyde and post-fixed using osmium tetroxide. Ultra-thin sections were cut and observed using TEM. The white arrows show vacuolar membrane and black arrows indicate bacteria. One representative micrographs out of three independent preparations.

In addition, human macrophages infected with *F. novicida* at MOI 10 at 15 min after infection and FCP within fractions were analyzed by transmission electron microscopy. At 15 min after infection bacteria were enclosed in intact phagosomes of infected hMDMs (Figure 6A). Low magnification TEM image of the FCP enriched fraction demonstrate the purity and small vesicle free fraction (Figure 6B). High magnification TEM image of the FCP enriched fraction revealed that single bacteria surrounded by per one-layer membrane were present (Figure 6C).

### Integrity of the FCP membrane after isolation

**Figure 7:**
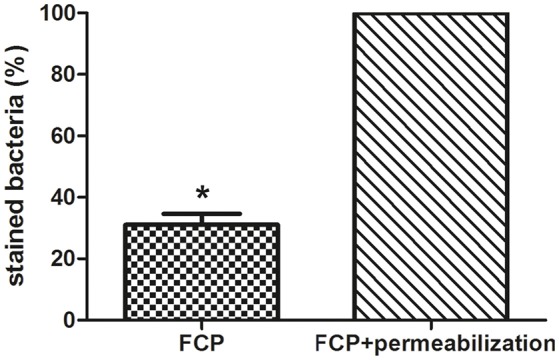
The analysis of FCP membrane integrity. Quantification of integrity of phagosomal membrane in the fraction with the highest number of bacteria. The percent of bacteria after staining with PI and analyses by fluorescence microscopy. Three coverslips were analyzed per fraction and STDEV were calculated. Student's *t*-test was used to determine statistical significance with ^*^P < 0.001.

The integrity of phagosomal membrane of isolated FCP was tested using PI by fluorescence microscopy. The fractions with the highest number of bacteria were used for this study. As a control sample, the isolated phagosomal fraction was permeabilized in order to allow the PI to penetrate inside the phagosome and stain the bacteria. This was considered as 100% of stained bacteria. Three coverslips were analyzed and the total stained bacteria in each sample were counted. This approach provided valuable information about the quality of the isolated FCP. Our results show that FCP membrane is intact on ~70% of the isolated phagosomal fractions (*P* = 0.000001; Figure 7).

## Discussion

It is essential for *Francisella* to replicate within host cells to successfully establish an infection and cause disease. Escape from the phagosome is an important step in *Francisella* life cycle since mutants deficient in escape are unable to cause productive infection (Chong et al., 2012). After invasion of the host cell, *Francisella* forms an endocytic membrane-bound phagosome. Bacteria must disrupt this phagosome and dislocate to the cytosol in order to replicate and spread from cell to cell (Clemens et al., 2004; Santic et al., 2005). However, these short-lived vacuoles can interact with the host vesicular trafficking, and can have important role for virulence and pathogenesis of *Francisella*. Due to importance of the phagosome in pathogenesis of tularemia we established a method for purification of FCP from infected human monocyte derived macrophages. To perform the separation of phagosome from infected cells, large cell number is necessary, making it challenging to use these approaches with human primary cells. For that reason, we pulled together the blood from different donors to obtain the necessary number of human macrophages. To ensure that we do not co-isolate the extracellular bacteria, intensive washing steps were included. In contrast to previously published protocols, the dextran coated iron particle were used for elimination of lysosomal and endosomal compartments prior infection (Shevchuk et al., 2009; Shevchuk and Steinert, 2013). With the use of confocal microscopy, we followed the trafficking of dextran-tetramethylrhodamine iron particles within hMDMs. The markers for early and late endosomes as well as lysosomes were used to document which endosomal compartments are labeled by colloidal iron. This is due to the specificity and rapidity of infection in comparison to other intracellular pathogens (Chong and Celli, 2010; Santic et al., 2010). Some of the most interesting aspects of phagosome maturation depend on the ability of intracellular pathogens to bypass the normal maturation process. Attempts to purify these compartments represents a challenge when classical organelle enrichment techniques are used. To resolve this problem a combination of classical and improved methods for enrichment and pre-fractionation must be used. In previous published methods for isolation of *Legionella*-containing vacuole the 5–30% OptiPrep gradient was used (Shevchuk et al., 2009; Shevchuk and Steinert, 2013). During the establishment of phagosome isolation from *Dictyostelium discoideum* cells infected with *F. novicida*, different concentration of OptiPrep were tested (5–30, 5–35, 10–40, and 10–45%, data not shown). Optimization of OptiPrep gradient for successful isolation of FCP showed that the best separation of FCP was when OptiPrep gradient 10–45% was used. In addition, the efficient removal of contaminants in the method of vacuole isolation is very important to achieve. Therefore, in order to separate two organelles with close density, mitochondria and FCP, we utilized an enzyme of the citrate cycle, the succinate dehydrogenase, located in the inner mitochondrial membrane. The INT added to the PNS is converted to formazan and increased the density of those organelles (Munujos et al., 1993). This phenomenon was used for separation of mitochondria from FCPs by discontinues ultracentrifugation method. In addition, Rab5 and EEA1, as markers for early endosomal compartment, were used to indicate the presence of FCP in OptiPrep fractions that was void of mitochondrial marker, AIF.

Isolation of bacteria-containing vacuoles (BCV) is of key importance for the understanding of these compartments, but technically is very challenging. During recent years, different groups have developed different protocols for isolation of BCV (reviewed in Herweg et al., 2015). The bacteria subvert endomembrane trafficking around the BCV and the communication around BCV and other host cell organelles has been described (Gagnon et al., 2002; Touret et al., 2005; Santos et al., 2015; Santos and Enninga, 2016).

Protocols for isolation of pathogen-containing vacuoles are based on subcellular/organelle fractionation based on physicochemical properties (Howe and Heinzen, 2008; He et al., 2012; Cheng et al., 2014). These protocols combine confocal fluorescence microscopy, Western blot and electron microscopy techniques providing the characterization of host cell compartments after infection with different intracellular pathogens. In addition, isolation of BCV can be based on immuno-affinity purification (Urwyler et al., 2010; Hoffmann et al., 2013; Vorwerk et al., 2015) or on FACS single cell enrichment by sorting bacteria and lysed host cells organelles (Becker et al., 2006; Pfortner et al., 2013; Surmann et al., 2014). Separation principles have been applied for isolation of latex bead-phagosomes from macrophages (Desjardins et al., 1994) and *Dictyostelium* (Gotthardt et al., 2002). In the protocol for purification of *Legionella*-containing vacuole (LCV) from infected *D. discoideum* (Shevchuk et al., 2009; Urwyler et al., 2010; Finsel et al., 2013; Shevchuk and Steinert, 2013), or murine macrophage-like RAW 264.7 (Hoffmann et al., 2014) the immuno-magnetic separation using an anti-SidC antibody was performed, followed by 10–35% Histodenz density gradient centrifugation. Others established protocol for LCV isolation from U937 macrophages using 55–65% gradient (Bruckert and Abu Kwaik, 2015). Others and our studies show that the integrity of phagosome membrane is often compromised during the early time point of infection with *Francisella* (Santic et al., 2008; Chong et al., 2012; Ozanic et al., 2015; Rowe and Huntley, 2015). Besides the electron microscopy methods, fluorescence microscopy could be valuable method to check the integrity of the phagosomal membrane after its isolation (Lonnbro et al., 2008; Hoffmann et al., 2013, 2014; Bruckert and Abu Kwaik, 2015). Results from this study show that the phagosomal membrane is highly conserved 15 min after infection of hMDMs and only 30% of the analyzed fraction show some damage of the FCP. The FCP is presumably intact within 30 min after infection.

Many intracellular bacteria reside and replicate inside phagosomal compartment making protocol for vacuolar isolation easy to apply. In contrast, some intracellular pathogens show ability to escape from phagosome to directly use the cytoplasm as their replicative habitat (Ray et al., 2009). *Francisella* resides in phagosomes ~5–30 min after infection, making it more challenging for isolation of the phagosome from infected macrophages. After cytoplasmic replication, *Francisella* re-enters the endocytic pathways by autophagy (Checroun et al., 2006; Jones et al., 2012), and bacteria are found in autophagosomes by 24 h after infection. Our established method for isolation of FCP could be applied for the isolation of autophagosomes as well. The protocol presented here will enable future proteomic analyses analysis of those delicate intracellular compartments.

## Author contributions

VM, OS, and MiS contributed in isolation of the vacuole and writing. MM and AJ participated in Western blot analyses. MO participated in electron microscopy analyses. MaS and YA participated in fluorescence analyses and writing.

### Conflict of interest statement

The authors declare that the research was conducted in the absence of any commercial or financial relationships that could be construed as a potential conflict of interest.

## References

[B1] Alvarez-Dominguez C. Barbieri A. M. Beron W. Wandinger-Ness A. Stahl P. D. (1996). Phagocytosed live *Listeria monocytogenes* influences Rab5-regulated *in vitro* phagosome-endosome fusion. J. Biol. Chem. 271, 13834–13843. 10.1074/jbc.271.23.13834 8662791

[B2] Anthony L. D. Burke R. D. Nano F. E. (1991). Growth of *Francisella* spp. in rodent macrophages. Infect. Immun. 59, 3291–3296. 1879943 10.1128/iai.59.9.3291-3296.1991 PMC258167

[B3] Anthony L. S. Gu M. Z. Cowley S. C. Leung W. W. Nano F. E. (1991). Transformation and allelic replacement in *Francisella* spp. J. Gen. Microbiol. 137, 2697–2703. 10.1099/00221287-137-12-2697 1791425

[B4] Barker J. R. Chong A. Wehrly T. D. Yu J. J. Rodriguez S. A. Liu J.. (2009). The *Francisella tularensis* pathogenicity island encodes a secretion system that is required for phagosome escape and virulence. Mol. Microbiol. 74, 1459–1470. 10.1111/j.1365-2958.2009.06947.x 20054881 PMC2814410

[B5] Becker D. Selbach M. Rollenhagen C. Ballmaier M. Meyer T. F. Mann M.. (2006). Robust Salmonella metabolism limits possibilities for new antimicrobials. Nature 440, 303–307. 10.1038/nature04616 16541065

[B6] Ben Nasr A. Haithcoat J. Masterson J. E. Gunn J. S. Eaves-Pyles T. Klimpel G. R. (2006). Critical role for serum opsonins and complement receptors CR3 (CD11b/CD18) and CR4 (CD11c/CD18) in phagocytosis of *Francisella tularensis* by human dendritic cells (DC): uptake of *Francisella* leads to activation of immature DC and intracellular survival of the bacteria. J. Leukoc. Biol. 80, 774–786. 10.1189/jlb.1205755 16857732

[B7] Birdsell D. N. Stewart T. Vogler A. J. Lawaczeck E. Diggs A. Sylvester T. L.. (2009). *Francisella tularensis* subsp. novicida isolated from a human in Arizona. BMC Res. Notes 2:223. 10.1186/1756-0500-2-223 19895698 PMC2780447

[B8] Bonquist L. Lindgren H. Golovliov I. Guina T. Sjostedt A. (2008). MglA and Igl proteins contribute to the modulation of *Francisella tularensis* live vaccine strain-containing phagosomes in murine macrophages. Infect. Immun. 76, 3502–3510. 10.1128/IAI.00226-08 18474647 PMC2493230

[B9] Broms J. E. Meyer L. Sun K. Lavander M. Sjostedt A. (2012). Unique substrates secreted by the type VI secretion system of *Francisella tularensis* during intramacrophage infection. PLoS ONE 7:e50473. 10.1371/journal.pone.0050473 23185631 PMC3502320

[B10] Broms J. E. Sjostedt A. Lavander M. (2010). The role of the *Francisella Tularensis* pathogenicity island in type VI secretion, intracellular survival, and modulation of host cell signaling. Front. Microbiol. 1:136. 10.3389/fmicb.2010.00136 21687753 PMC3109350

[B11] Bruckert W. M. Abu Kwaik Y. (2015). Complete and ubiquitinated proteome of the Legionella-containing vacuole within human macrophages. J. Proteome Res. 14, 236–248. 10.1021/pr500765x 25369898 PMC4286187

[B12] Buddingh G. J. Womack F. C. (1941). Observations on the infection of chick embryos with Bacterium Tularense, Brucella, and Pasteurella Pestis. J. Exp. Med. 74, 213–222. 10.1084/jem.74.3.213 19871129 PMC2135183

[B13] Camejo A. Carvalho F. Reis O. Leitao E. Sousa S. Cabanes D. (2011). The arsenal of virulence factors deployed by *Listeria monocytogenes* to promote its cell infection cycle. Virulence 2, 379–394. 10.4161/viru.2.5.17703 21921683

[B14] Celli J. Zahrt T. C. (2013). Mechanisms of *Francisella tularensis* intracellular pathogenesis. Cold Spring Harb. Perspect. Med. 3:a010314. 10.1101/cshperspect.a010314 23545572 PMC3683997

[B15] Checroun C. Wehrly T. D. Fischer E. R. Hayes S. F. Celli J. (2006). Autophagy-mediated reentry of *Francisella tularensis* into the endocytic compartment after cytoplasmic replication. Proc. Natl. Acad. Sci. U.S.A. 103, 14578–14583. 10.1073/pnas.0601838103 16983090 PMC1600002

[B16] Cheng Y. Liu Y. Wu B. Zhang J. Z. Gu J. Liao Y. L.. (2014). Proteomic analysis of the *Ehrlichia chaffeensis* phagosome in cultured DH82 cells. PLoS ONE 9:e88461. 10.1371/journal.pone.0088461 24558391 PMC3928192

[B17] Chong A. Celli J. (2010). The francisella intracellular life cycle: toward molecular mechanisms of intracellular survival and proliferation. Front. Microbiol. 1:138. 10.3389/fmicb.2010.00138 21687806 PMC3109316

[B18] Chong A. Child R. Wehrly T. D. Rockx-Brouwer D. Qin A. Mann B. J.. (2013). Structure-function analysis of DipA, a *Francisella tularensis* virulence Factor required for intracellular replication. PLoS ONE 8:e67965. 10.1371/journal.pone.0067965 23840797 PMC3694160

[B19] Chong A. Wehrly T. D. Child R. Hansen B. Hwang S. Virgin H. W.. (2012). Cytosolic clearance of replication-deficient mutants reveals *Francisella tularensis* interactions with the autophagic pathway. Autophagy 8, 1342–1356. 10.4161/auto.20808 22863802 PMC3442881

[B20] Chong A. Wehrly T. D. Nair V. Fischer E. R. Barker J. R. Klose K. E.. (2008). The early phagosomal stage of *Francisella tularensis* determines optimal phagosomal escape and Francisella pathogenicity island protein expression. Infect. Immun. 76, 5488–5499. 10.1128/IAI.00682-08 18852245 PMC2583578

[B21] Clemens D. L. Lee B. Y. Horwitz M. A. (2004). Virulent and avirulent strains of *Francisella tularensis* prevent acidification and maturation of their phagosomes and escape into the cytoplasm in human macrophages. Infect. Immun. 72, 3204–3217. 10.1128/IAI.72.6.3204-3217.2004 15155622 PMC415696

[B22] Clemens D. L. Lee B. Y. Horwitz M. A. (2005). *Francisella tularensis* enters macrophages via a novel process involving pseudopod loops. Infect. Immun. 73, 5892–5902. 10.1128/IAI.73.9.5892-5902.2005 16113308 PMC1231130

[B23] Clemens D. L. Lee B. Y. Horwitz M. A. (2009). *Francisella tularensis* phagosomal escape does not require acidification of the phagosome. Infect. Immun. 77, 1757–1773. 10.1128/IAI.01485-08 19237528 PMC2681761

[B24] Desjardins M. Huber L. A. Parton R. G. Griffiths G. (1994). Biogenesis of phagolysosomes proceeds through a sequential series of interactions with the endocytic apparatus. J. Cell Biol. 124, 677–688. 10.1083/jcb.124.5.677 8120091 PMC2119957

[B25] Duclos S. Diez R. Garin J. Papadopoulou B. Descoteaux A. Stenmark H.. (2000). Rab5 regulates the kiss and run fusion between phagosomes and endosomes and the acquisition of phagosome leishmanicidal properties in RAW 264.7 macrophages. J. Cell Sci. 113(Pt 19), 3531–3541. 10984443 10.1242/jcs.113.19.3531

[B26] Finsel I. Hoffmann C. Hilbi H. (2013). Immunomagnetic purification of fluorescent Legionella-containing vacuoles. Methods Mol. Biol. 983, 431–443. 10.1007/978-1-62703-302-2_24 23494322

[B27] Fortier A. H. Green S. J. Polsinelli T. Jones T. R. Crawford R. M. Leiby D. A.. (1994). Life and death of an intracellular pathogen: *Francisella tularensis* and the macrophage. Immunol. Ser. 60, 349–361. 8251580

[B28] Gagnon E. Duclos S. Rondeau C. Chevet E. Cameron P. H. Steele-Mortimer O.. (2002). Endoplasmic reticulum-mediated phagocytosis is a mechanism of entry into macrophages. Cell 110, 119–131. 10.1016/S0092-8674(02)00797-3 12151002

[B29] Golovliov I. Baranov V. Krocova Z. Kovarova H. Sjostedt A. (2003). An attenuated strain of the facultative intracellular bacterium *Francisella tularensis* can escape the phagosome of monocytic cells. Infect. Immun. 71, 5940–5950. 10.1128/IAI.71.10.5940-5950.2003 14500514 PMC201066

[B30] Gotthardt D. Warnatz H. J. Henschel O. Bruckert F. Schleicher M. Soldati T. (2002). High-resolution dissection of phagosome maturation reveals distinct membrane trafficking phases. Mol. Biol. Cell 13, 3508–3520. 10.1091/mbc.E02-04-0206 12388753 PMC129962

[B31] Hare R. F. Hueffer K. (2014). *Francisella novicida* pathogenicity island encoded proteins were secreted during infection of macrophage-like cells. PLoS ONE 9:e105773. 10.1371/journal.pone.0105773 25158041 PMC4144950

[B32] He Y. Li W. Liao G. Xie J. (2012). Mycobacterium tuberculosis-specific phagosome proteome and underlying signaling pathways. J. Proteome Res. 11, 2635–2643. 10.1021/pr300125t 22443300

[B33] Herweg J. A. Hansmeier N. Otto A. Geffken A. C. Subbarayal P. Prusty B. K.. (2015). Purification and proteomics of pathogen-modified vacuoles and membranes. Front. Cell. Infect. Microbiol. 5:48. 10.3389/fcimb.2015.00048 26082896 PMC4451638

[B34] Hoffmann C. Finsel I. Hilbi H. (2013). Pathogen vacuole purification from legionella-infected amoeba and macrophages. Methods Mol. Biol. 954, 309–321. 10.1007/978-1-62703-161-5_18 23150404

[B35] Hoffmann C. Finsel I. Otto A. Pfaffinger G. Rothmeier E. Hecker M.. (2014). Functional analysis of novel Rab GTPases identified in the proteome of purified Legionella-containing vacuoles from macrophages. Cell. Microbiol. 16, 1034–1052. 10.1111/cmi.12256 24373249

[B36] Howe D. Heinzen R. A. (2008). Fractionation of the *Coxiella burnetii* parasitophorous vacuole. Methods Mol. Biol. 445, 389–406. 10.1007/978-1-59745-157-4_25 18425464 PMC2679505

[B37] Isberg R. R. O'connor T. J. Heidtman M. (2009). The *Legionella pneumophila* replication vacuole: making a cosy niche inside host cells. Nat. Rev. Microbiol. 7, 13–24. 10.1038/nrmicro1967 19011659 PMC2631402

[B38] Jahraus A. Tjelle T. E. Berg T. Habermann A. Storrie B. Ullrich O.. (1998). *In vitro* fusion of phagosomes with different endocytic organelles from J774 macrophages. J. Biol. Chem. 273, 30379–30390. 10.1074/jbc.273.46.30379 9804802

[B39] Jones C. L. Napier B. A. Sampson T. R. Llewellyn A. C. Schroeder M. R. Weiss D. S. (2012). Subversion of host recognition and defense systems by *Francisella* spp. Microbiol. Mol. Biol. Rev. 76, 383–404. 10.1128/MMBR.05027-11 22688817 PMC3372254

[B40] Kagan J. C. Roy C. R. (2002). Legionella phagosomes intercept vesicular traffic from endoplasmic reticulum exit sites. Nat. Cell Biol. 4, 945–954. 10.1038/ncb883 12447391

[B41] Lauriano C. M. Barker J. R. Yoon S. S. Nano F. E. Arulanandam B. P. Hassett D. J.. (2004). MglA regulates transcription of virulence factors necessary for *Francisella tularensis* intraamoebae and intramacrophage survival. Proc. Natl. Acad. Sci. U.S.A. 101, 4246–4249. 10.1073/pnas.0307690101 15010524 PMC384726

[B42] Lindemann S. R. McLendon M. K. Apicella M. A. Jones B. D. (2007). An *in vitro* model system used to study adherence and invasion of *Francisella tularensis* live vaccine strain in nonphagocytic cells. Infect. Immun. 75, 3178–3182. 10.1128/IAI.01811-06 17339345 PMC1932879

[B43] Lindgren H. Golovliov I. Baranov V. Ernst R. K. Telepnev M. Sjostedt A. (2004). Factors affecting the escape of *Francisella tularensis* from the phagolysosome. J. Med. Microbiol. 53, 953–958. 10.1099/jmm.0.45685-0 15358816

[B44] Llewellyn A. C. Jones C. L. Napier B. A. Bina J. E. Weiss D. S. (2011). Macrophage replication screen identifies a novel Francisella hydroperoxide resistance protein involved in virulence. PLoS ONE 6:e24201. 10.1371/journal.pone.0024201 21915295 PMC3167825

[B45] Lonnbro P. Nordenfelt P. Tapper H. (2008). Isolation of bacteria-containing phagosomes by magnetic selection. BMC Cell Biol. 9:35. 10.1186/1471-2121-9-35 18588680 PMC2453110

[B46] Malik-Kale P. Jolly C. E. Lathrop S. Winfree S. Luterbach C. Steele-Mortimer O. (2011). Salmonella - at home in the host cell. Front. Microbiol. 2:125. 10.3389/fmicb.2011.00125 21687432 PMC3109617

[B47] McCaffrey R. L. Allen L. A. (2006). *Francisella tularensis* LVS evades killing by human neutrophils via inhibition of the respiratory burst and phagosome escape. J. Leukoc. Biol. 80, 1224–1230. 10.1189/jlb.0406287 16908516 PMC1828114

[B48] Meyer L. Broms J. E. Liu X. Rottenberg M. E. Sjostedt A. (2015). Microinjection of *Francisella tularensis* and *Listeria monocytogenes* reveals the importance of bacterial and host factors for successful replication. Infect. Immun. 83, 3233–3242. 10.1128/IAI.00416-15 26034213 PMC4496618

[B49] Moreau G. B. Mann B. J. (2013). Adherence and uptake of Francisella into host cells. Virulence 4, 826–832. 10.4161/viru.25629 23921460 PMC3925714

[B50] Munujos P. Coll-Canti J. Gonzalez-Sastre F. Gella F. J. (1993). Assay of succinate dehydrogenase activity by a colorimetric-continuous method using iodonitrotetrazolium chloride as electron acceptor. Anal. Biochem. 212, 506–509. 10.1006/abio.1993.1360 8214593

[B51] Nano F. E. Zhang N. Cowley S. C. Klose K. E. Cheung K. K. Roberts M. J.. (2004). A *Francisella tularensis* pathogenicity island required for intramacrophage growth. J. Bacteriol. 186, 6430–6436. 10.1128/JB.186.19.6430-6436.2004 15375123 PMC516616

[B52] Napier B. A. Meyer L. Bina J. E. Miller M. A. Sjostedt A. Weiss D. S. (2012). Link between intraphagosomal biotin and rapid phagosomal escape in Francisella. Proc. Natl. Acad. Sci. U.S.A. 109, 18084–18089. 10.1073/pnas.1206411109 23071317 PMC3497780

[B53] Ozanic M. Marecic V. Abu Kwaik Y. Santic M. (2015). The divergent intracellular lifestyle of *Francisella tularensis* in evolutionarily distinct host cells. PLoS Pathog. 11:e1005208. 10.1371/journal.ppat.1005208 26633893 PMC4669081

[B54] Ozanic M. Marecic V. Lindgren M. Sjostedt A. Santic M. (2016). Phenotypic characterization of the *Francisella tularensis* DeltapdpC and DeltaiglG mutants. Microbes Infect. 18, 768–776. 10.1016/j.micinf.2016.07.006 27477000

[B55] Pfortner H. Wagner J. Surmann K. Hildebrandt P. Ernst S. Bernhardt J.. (2013). A proteomics workflow for quantitative and time-resolved analysis of adaptation reactions of internalized bacteria. Methods 61, 244–250. 10.1016/j.ymeth.2013.04.009 23643866

[B56] Qin A. Mann B. J. (2006). Identification of transposon insertion mutants of *Francisella tularensis* tularensis strain Schu S4 deficient in intracellular replication in the hepatic cell line HepG2. BMC Microbiol. 6:69. 10.1186/1471-2180-6-69 16879747 PMC1557513

[B57] Ray K. Bobard A. Danckaert A. Paz-Haftel I. Clair C. Ehsani S.. (2010). Tracking the dynamic interplay between bacterial and host factors during pathogen-induced vacuole rupture in real time. Cell. Microbiol. 12, 545–556. 10.1111/j.1462-5822.2010.01428.x 20070313

[B58] Ray K. Marteyn B. Sansonetti P. J. Tang C. M. (2009). Life on the inside: the intracellular lifestyle of cytosolic bacteria. Nat. Rev. Microbiol. 7, 333–340. 10.1038/nrmicro2112 19369949

[B59] Rodriguez-Paris J. M. Nolta K. V. Steck T. L. (1993). Characterization of lysosomes isolated from *Dictyostelium discoideum* by magnetic fractionation. J. Biol. Chem. 268, 9110–9116. 7682559

[B60] Rowe H. M. Huntley J. F. (2015). From the outside-in: the *Francisella tularensis* envelope and virulence. Front. Cell. Infect. Microbiol. 5:94. 10.3389/fcimb.2015.00094 26779445 PMC4688374

[B61] Santic M. Akimana C. Asare R. Kouokam J. C. Atay S. Kwaik Y. A. (2009). Intracellular fate of *Francisella tularensis* within arthropod-derived cells. Environ. Microbiol. 11, 1473–1481. 10.1111/j.1462-2920.2009.01875.x 19220402

[B62] Santic M. Al-Khodor S. Abu Kwaik Y. (2010). Cell biology and molecular ecology of *Francisella tularensis*. Cell. Microbiol. 12, 129–139. 10.1111/j.1462-5822.2009.01400.x 19863554

[B63] Santic M. Asare R. Skrobonja I. Jones S. Abu Kwaik Y. (2008). Acquisition of the vacuolar ATPase proton pump and phagosome acidification are essential for escape of *Francisella tularensis* into the macrophage cytosol. Infect. Immun. 76, 2671–2677. 10.1128/IAI.00185-08 18390995 PMC2423071

[B64] Santic M. Molmeret M. Abu Kwaik Y. (2005). Modulation of biogenesis of the *Francisella tularensis* subsp. novicida-containing phagosome in quiescent human macrophages and its maturation into a phagolysosome upon activation by IFN-gamma. Cell. Microbiol. 7, 957–967. 10.1111/j.1462-5822.2005.00529.x 15953028

[B65] Santos J. C. Enninga J. (2016). At the crossroads: communication of bacteria-containing vacuoles with host organelles. Cell. Microbiol. 18, 330–339. 10.1111/cmi.12567 26762760

[B66] Santos S. B. Carvalho C. Azeredo J. Ferreira E. C. (2015). Correction: population dynamics of a salmonella lytic phage and its host: implications of the host bacterial growth rate in modelling. PLoS ONE 10:e0136007. 10.1371/journal.pone.0136007 26305566 PMC4549271

[B67] Shepard C. C. (1959). Nonacid-fast bacteria and HeLa cells: their uptake and subsequent intracellular growth. J. Bacteriol. 77, 701–714. 13664649 10.1128/jb.77.6.701-714.1959 PMC290452

[B68] Shevchuk O. Batzilla C. Hagele S. Kusch H. Engelmann S. Hecker M.. (2009). Proteomic analysis of Legionella-containing phagosomes isolated from Dictyostelium. Int. J. Med. Microbiol. 299, 489–508. 10.1016/j.ijmm.2009.03.006 19482547

[B69] Shevchuk O. Steinert M. (2013). Isolation of pathogen-containing vacuoles. Methods Mol. Biol. 983, 419–429. 10.1007/978-1-62703-302-2_23 23494321

[B70] Shin S. Roy C. R. (2008). Host cell processes that influence the intracellular survival of Legionella pneumophila. Cell. Microbiol. 10, 1209–1220. 10.1111/j.1462-5822.2008.01145.x 18363881

[B71] Steele S. Brunton J. Ziehr B. Taft-Benz S. Moorman N. Kawula T. (2013). *Francisella tularensis* harvests nutrients derived via ATG5-independent autophagy to support intracellular growth. PLoS Pathog. 9:e1003562. 10.1371/journal.ppat.1003562 23966861 PMC3744417

[B72] Steele-Mortimer O. (2008). The Salmonella-containing vacuole - Moving with the times. Curr. Opin. Microbiol. 11, 38–45. 10.1016/j.mib.2008.01.002 18304858 PMC2577838

[B73] Surmann K. Michalik S. Hildebrandt P. Gierok P. Depke M. Brinkmann L.. (2014). Comparative proteome analysis reveals conserved and specific adaptation patterns of *Staphylococcus aureus* after internalization by different types of human non-professional phagocytic host cells. Front. Microbiol. 5:392. 10.3389/fmicb.2014.00392 25136337 PMC4117987

[B74] Tamilselvam B. Daefler S. (2008). Francisella targets cholesterol-rich host cell membrane domains for entry into macrophages. J. Immunol. 180, 8262–8271. 10.4049/jimmunol.180.12.8262 18523292

[B75] Telepnev M. Golovliov I. Sjostedt A. (2005). *Francisella tularensis* LVS initially activates but subsequently down-regulates intracellular signaling and cytokine secretion in mouse monocytic and human peripheral blood mononuclear cells. Microb. Pathog. 38, 239–247. 10.1016/j.micpath.2005.02.003 15925273

[B76] Touret N. Paroutis P. Terebiznik M. Harrison R. E. Trombetta S. Pypaert M.. (2005). Quantitative and dynamic assessment of the contribution of the ER to phagosome formation. Cell 123, 157–170. 10.1016/j.cell.2005.08.018 16213220

[B77] Urwyler S. Finsel I. Ragaz C. Hilbi H. (2010). Isolation of Legionella-containing vacuoles by immuno-magnetic separation. Curr. Protoc. Cell Biol. Chapter 3, Unit 3.34. 10.1002/0471143030.cb0334s46 20235103

[B78] Vergne I. Chua J. Singh S. B. Deretic V. (2004). Cell biology of mycobacterium tuberculosis phagosome. Annu. Rev. Cell Dev. Biol. 20, 367–394. 10.1146/annurev.cellbio.20.010403.114015 15473845

[B79] Vorwerk S. Krieger V. Deiwick J. Hensel M. Hansmeier N. (2015). Proteomes of host cell membranes modified by intracellular activities of *Salmonella enterica*. Mol. Cell. Proteomics 14, 81–92. 10.1074/mcp.M114.041145 25348832 PMC4288265

[B80] Wehrly T. D. Chong A. Virtaneva K. Sturdevant D. E. Child R. Edwards J. A.. (2009). Intracellular biology and virulence determinants of *Francisella tularensis* revealed by transcriptional profiling inside macrophages. Cell. Microbiol. 11, 1128–1150. 10.1111/j.1462-5822.2009.01316.x 19388904 PMC2746821

[B81] Wetzel M. G. Korn E. D. (1969). Phagocytosis of latex beads by *Acahamoeba castellanii* (Neff). 3. Isolation of the phagocytic vesicles and their membranes. J. Cell Biol. 43, 90–104. 10.1083/jcb.43.1.90 4309954 PMC2107834

